# Fat-poor angiomyolipoma with cyst-like changes mimicking a cystic renal cell carcinoma: a case report

**DOI:** 10.1186/s12957-015-0677-4

**Published:** 2015-08-19

**Authors:** Yuki Kobari, Toshio Takagi, Tsunenori Kondo, Hidekazu Tachibana, Shoichi Iida, Yu Nishina, Kenji Omae, Satoru Morita, Tomoko Yamamoto, Junpei Iizuka, Yoji Nagashima, Kazunari Tanabe

**Affiliations:** Department of Urology, Tokyo Women’s Medical University, 8-1, Kawada-cho, Shinjuku-ku, Tokyo, 162-8666 Japan; Department of Diagnostic Imaging and Nuclear Medicine, Tokyo Women’s Medical University, Tokyo, Japan; Department of Surgical Pathology, Tokyo Women’s Medical University, Tokyo, Japan

**Keywords:** Fat-poor angiomyolipoma, Cystic renal cell carcinoma, Renal biopsy

## Abstract

Angiomyolipoma is a common benign renal tumor. It is typically composed of adipose tissue and hence is easily diagnosed by using imaging methods such as ultrasonography, computed tomography, and magnetic resonance imaging. However, it is difficult to differentiate an atypical angiomyolipoma such as a fat-poor angiomyolipoma from a malignant tumor by using these imaging methods. We report a case of a fat-poor angiomyolipoma with cyst-like changes in a 35-year-old man. The angiomyolipoma was initially suspected to be a cystic renal cell carcinoma according to preoperative imaging studies. A 5-cm cystic tumor with an enhanced septal wall and exophytic formation was present in the middle section of the left kidney. The patient underwent partial nephrectomy. Pathological findings showed necrosis and hematoma in almost the entire lesion, with a small amount of adipose and muscle tissue. Finally, a fat-poor angiomyolipoma was diagnosed.

## Background

Angiomyolipoma (AML) is one of the most common benign solid triphasic renal tumors and is composed of varying amounts of dysmorphic blood vessels, smooth muscles, and mature adipose tissue [1, 2]. As most AMLs contain substantial amounts of adipose tissue, it is usually diagnosed by using computed tomography (CT) or magnetic resonance imaging (MRI), both of which identify the characteristic imaging features of fat cells in the mass. AMLs that can be diagnosed on imaging are called “classic” AMLs. On the other hand, different types of AML, which include a heterogeneous group of neoplasms with variable clinical behavior, radiology, and pathology, also exist. In particular, some triphasic AMLs contain very few fat cells, which cannot be detected on imaging. These are called fat-poor AMLs and are sometimes mistaken for renal cancers [3–6]. As already mentioned, the cystic renal mass contains few or no fat cells, and it is categorized as a type of fat-poor AML known as an AML with epithelial cysts. Although AML is a benign tumor, a few cases of epithelioid AML develop malignant clinical courses [7, 8]. Herein, we report a case of a fat-poor AML with cyst-like changes.

## Case presentation

A 35-year-old-Japanese man was referred to our department because of left back pain. He did not have any relevant medical or family history.

**Fig. 1:**
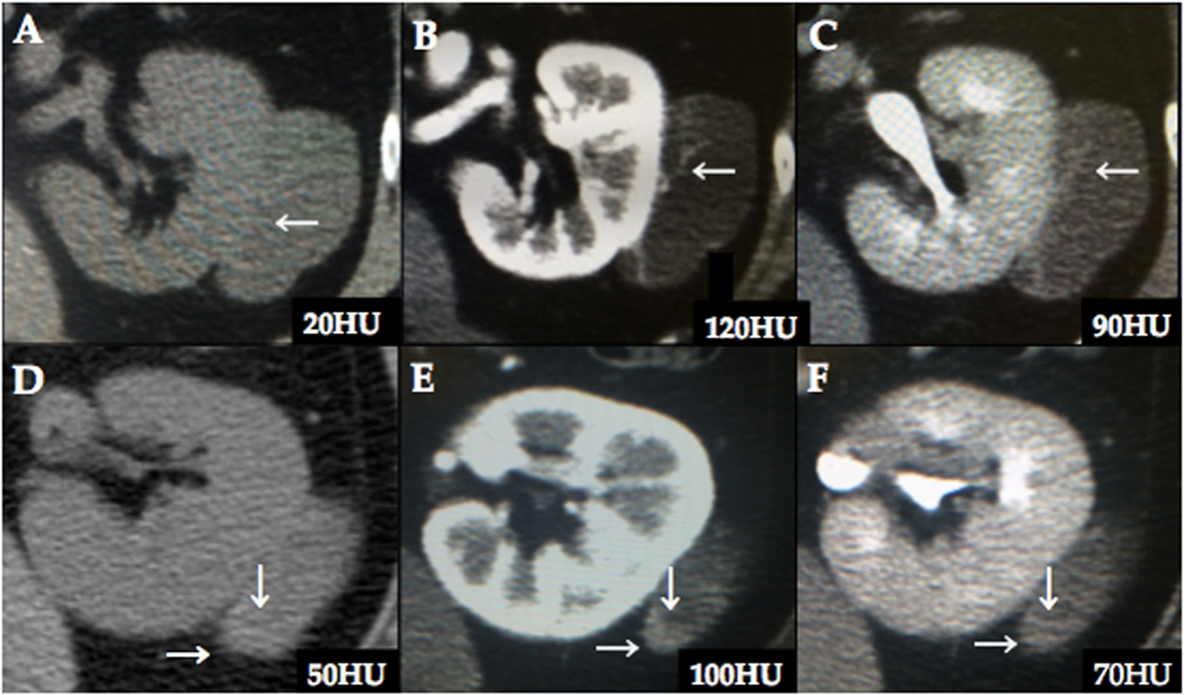
Preoperative computed tomography. The renal mass appears mainly as a hypodense area (**a**) with a partially hyperdense area (**d**) on unenhanced computed tomography. The hyperdense area and septa are enhanced in the early phase (**b, e**) and washed out in the late phase (**c, f**). White arrows show the area where CT attenuation value is calculated

**Fig. 2:**
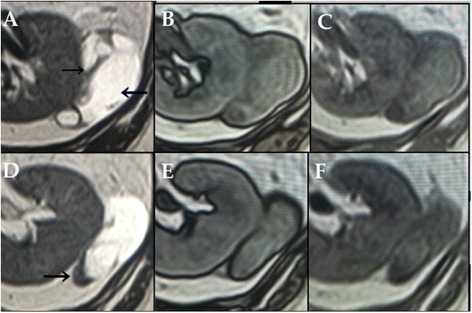
Preoperative magnetic resonance imaging (MRI). On T2-weighted MRI, the cystic mass and septal wall appear as hyperintense and hypointense areas, respectively. Moreover, the continuity between the renal parenchyma and the cystic mass is not clear (**a, d – black arrows**). T1-weighted MRI does not show a signal change between the in-phase (**c, f**) and out-of-phase (**b, e**) images, indicating that the cystic mass has no adipose tissue

Ultrasonography showed a low-echoic cystic lesion in the left kidney. CT revealed a 5-cm cystic renal mass with a mixed compartment consisting of a mainly hypodense area with a partially hyperdense area in the middle pole of the left kidney. The hyperdense area and septa were enhanced in the early phase and washed out in the late phase (Fig. 1). On T2-weighted MRI, the cystic mass and septal wall appeared as hyperintense and hypointense areas, respectively. Moreover, the continuity between the renal parenchyma and the cystic mass was not clear. T1-weighted MRI showed no signal change between the in-phase and out-of-phase images, indicating that this cystic mass had no adipose tissue (Fig. 2). Finally, the patient was radiologically suspected to have a cystic renal cell carcinoma (Bosniak category IV) or a retroperitoneal tumor.

**Fig. 3:**
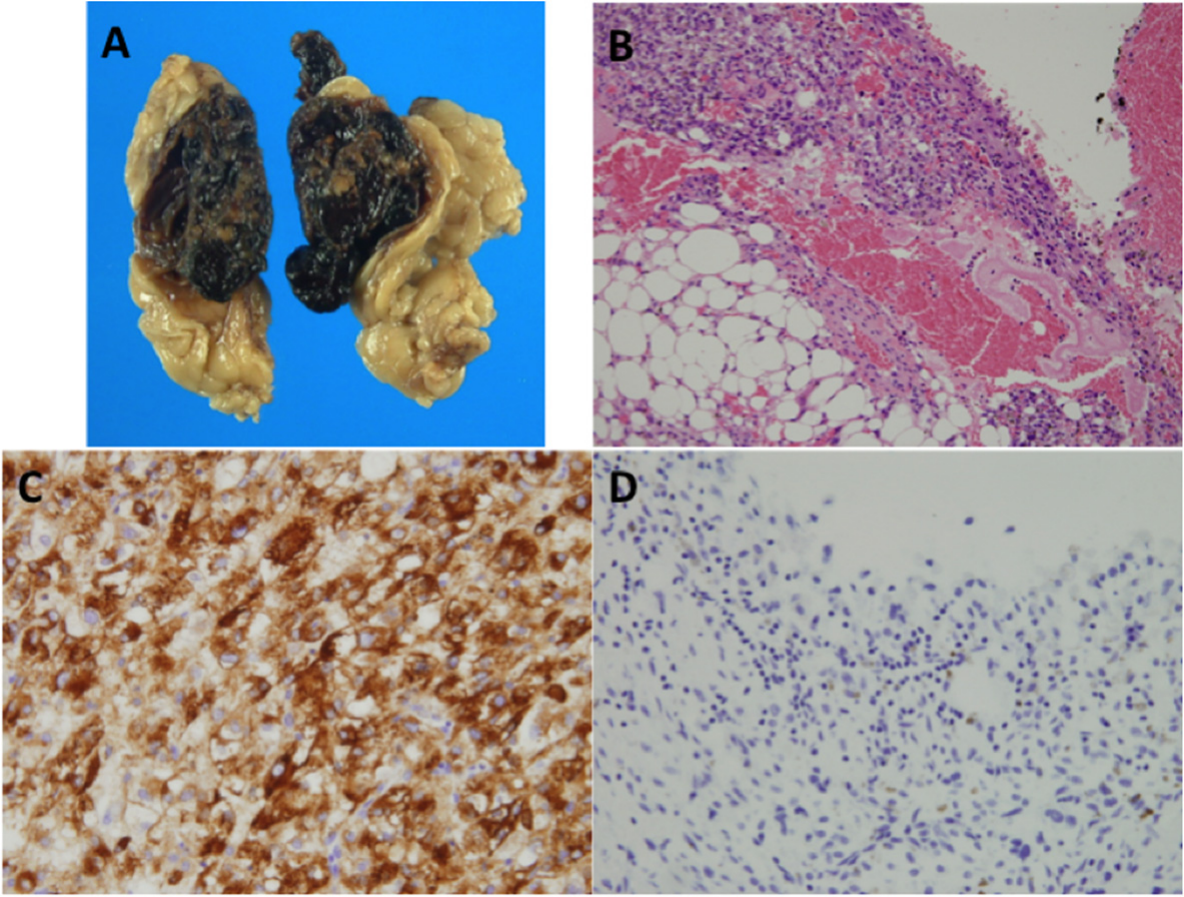
Pathological findings of the resected tumor. Grossly, the tumor was continuous with the renal capsule and adherent with the perirenal fat. The border is well demarcated. The cut surface is dark red with hemorrhage (**a**). Histologically, the tumor is mostly hemorrhagic with a less amount of spindle cells and fat in the septa (hematoxylin and eosin, original magnification ×100) (**b**). Immunohistochemically, the spindle-shaped cells are reactive with the human melanoma black-45 antibody, demonstrating the melanosome-associated antigen, (**c**) but negative for cytokeratin (antibody clone AE1/AE3). The cyst wall lacks epithelial lining, unlike angiomyolipoma with epithelial cyst (**d**)

The patient underwent partial nephrectomy. The tumor peeled away easily from the renal surface and adhered to the renal capsule with a very small area, suggesting that the tumor had originated in the kidney. The size of the surgical specimen was 4.0 × 4.0 × 2.5 cm, and macroscopic findings showed dark reddish spongiform contents rounded with capsule (Fig. 3a). Pathologically, the tumor showed predominance. Notably, the septa did not contain adipose tissue and smooth muscle-like spindle cells (Fig. 3b). Immunohistochemically, the spindle cells were positive for melanosome-associated antigen detected by the human melanoma black-45 antibody (Fig. 3c), smooth muscle actin, and S-100 protein, but negative for cytokeratin (AE1/AE3; Fig. 3d). The Ki-67 labeling index was less than 5 %. Accordingly, the tumor was diagnosed as a fat-poor AML causing a cystic change due to intratumoral hemorrhage.

### Discussion

Renal AML is a solid tumor that is encountered commonly in clinical practice [2]. AML is typically a solid triphasic tumor composed of varying amounts of the following three elements: dysmorphic blood vessels, smooth muscle, and mature adipose tissue. AMLs belong to the perivascular epithelioid cell tumor family in the 2002 World Health Organization classification [9, 10]. AMLs are usually asymptomatic and have no abnormal findings on medical examinations, but symptoms such as hematuria, pain, and mass palpation and signs such as retroperitoneal bleeding often occur when the tumor size is more than 4 cm [11–13]. AML is comparatively easy to diagnose through fat detection by using CT or MRI [2]. However, recent research has shown that some AMLs contain too little fat to be detected on unenhanced CT [14, 15] and that it is hard to distinguish some AMLs with cystic lesions from renal cell carcinoma because of intratumoral bleeding [16]. In the present case, it was difficult to diagnose AML because of a cyst-like formation and no fat tissue on preoperative imaging. Moreover, the tumor had the potential to be malignant owing to a slightly enhanced septal wall within the cystic area. Therefore, the patient underwent partial nephrectomy. The tumor was finally diagnosed as a “cyst-like change” rather than a renal cyst, suggesting that a hemorrhagic lesion from the AML had gradually expanded within the renal capsule and that it might have been diagnosed as a cystic mass on preoperative imaging. These implications were supported by the pathological findings of the hemorrhagic area and absence of cytokeratin in the cyst wall.

The term “AML with minimal fat” was reported for the first time in 1997 and accounts for approximately 5 % of all AMLs [17]. Various terms have been used for AMLs containing very little fat such as “lipid-poor AML” or “minimal fat AML.” Jinzaki et al. defined AMLs with no evidence of fat cells on unenhanced CT as fat-poor AMLs because of the pathology of these lesions, and they explained the presence of multiple subtypes of fat-poor AMLs to reduce readers’ confusion [1].

Some reports recommend a biopsy for tumors with an attenuation value of more than 40 Hounsfield units on unenhanced CT, and without fat cells on MRI, to differentiate AMLs from renal cell carcinoma [1, 18]. Our case was not suitable for a tumor biopsy because of the cyst-like formation.

We report a case of a fat-poor AML with cyst-like changes, which was difficult to differentiate from a cystic renal cell carcinoma by using preoperative imaging. The etiology of this cystic change from a very tiny tumor is relatively rare. Therefore, we present this case report.

## Conclusions

We report a case of a fat-poor AML with cyst-like changes, which was difficult to differentiate from a cystic renal cell carcinoma by using preoperative imaging.

## Consent

Written informed consent was obtained from the patient for publication of this case report and any accompanying images. A copy of the written consent is available for review by the Editor-in-Chief of this journal.

## Abbreviations

AML: angiomyolipoma
CT: computed tomography
MRI: magnetic resonance imaging
